# A Rare Presentation of Pseudo-Pneumoperitoneum Secondary to Chilaiditi Sign and Chilaiditi Syndrome in Two Pre-adolescent Females: A Case Series

**DOI:** 10.7759/cureus.48949

**Published:** 2023-11-17

**Authors:** Victoria Vazquez, Nikki Jones, Caren Ishikawa, Pankaj Watal, Syed Ali

**Affiliations:** 1 Graduate Medical Education, Nemours Children's Health System, Orlando, USA; 2 Radiology, Nemours Children's Health System, Orlando, USA; 3 Inpatient Pediatrics, Nemours Children's Hospital, Orlando, USA

**Keywords:** hepatodiaghramatic interpositioning of colon, pediatric abdominal pain, pseudo-pneumoperitoneum, chilaiditi’s sign, chilaiditi’s syndrome

## Abstract

Chilaiditi sign is defined as the interposition of the colon or small intestine between the liver and the right diaphragm in the absence of symptoms. Chilaiditi syndrome refers to the condition where the Chilaiditi sign is associated with symptoms including abdominal pain. In this series, we present the cases of two pre-pubescent patients with these rare conditions.

A 10-year-old female with a history of autism, IgA deficiency, and constipation presented for gastrointestinal studies due to weight loss and constipation. An abdominal X-ray revealed bowel gas under the right hemidiaphragm and colonic interposition between the diaphragm and the liver, raising concerns for the Chilaiditi sign. She underwent a bowel cleanout, with studies revealing colonic dysmotility and compartmentalization of the sigmoid colon and rectum with the absence of coloanal reflex.

A nine-year-old female with a history of constipation, developmental delay, and hypotonia presented with abdominal pain, vomiting, constipation, and decreased appetite. She also manifested tachypnea, abdominal distension, and abdominal tenderness, with an abdominal X-ray revealing a dilated colon interposed between the liver and diaphragm, confirming Chilaiditi syndrome. Prior gastrointestinal studies showed dilated and redundant sigmoid colon and dyssynergia. The treatment entailed rectal irrigations and catheter decompression, which led to the improvement of symptoms.

Conservative treatment is the treatment of choice for patients with Chilaiditi sign or Chilaiditi syndrome. It is important to distinguish Chilaiditi syndrome, a common cause of pseudo-pneumoperitoneum, from true pneumoperitoneum, as this diagnosis warrants immediate surgical intervention. Surgical treatment is indicated when there are signs of bowel obstruction or ischemia and for cases with recurrent Chilaiditi syndrome. Raising awareness about this condition is important to reduce the incidence of misdiagnosed surgical emergencies and resulting exploratory surgeries, as well as to avoid high-risk colonoscopies. Chilaiditi sign and Chilaiditi syndrome are relatively uncommon entities, and their prevalence is very rare in the pediatric population. Hence, we believe this case series will contribute to providing clinical awareness of these major complications and avoiding invasive interventions due to the inaccurate diagnosis of these conditions as pneumoperitoneum.

## Introduction

Chilaiditi sign refers to the interposition of the colon or small intestine between the liver and the right diaphragm in the absence of symptoms. The hepatodiaphragmatic interpositioning of the intestine was first described by Cantini in 1865; however, the condition is named after Demetrius Chilaiditi who reported the radiologic findings in 1910. It is usually an incidental finding in 0.025-0.28% of the general population and its incidence appears to rise with age. It is reportedly more common in males than females [1,2]. When the hepatodiaphragmatic interpositioning of the intestine presents with symptoms, it is called Chilaiditi syndrome. Common symptoms associated with Chilaiditi syndrome in children are abdominal pain, vomiting, abdominal distention, and constipation [3]. Although it is very rare in childhood, there have been cases reports of Chilaiditi syndrome and its signs in children as young as 10 days [4] to those aged 11 years [3], and there has been a surge in reports of this condition among children of both genders in the past 10 years [4-9].

This article was previously presented as a meeting abstract at the 2023 Nemours Children’s Health Research Week.

## Case presentation

Case 1

**Figure 1:**
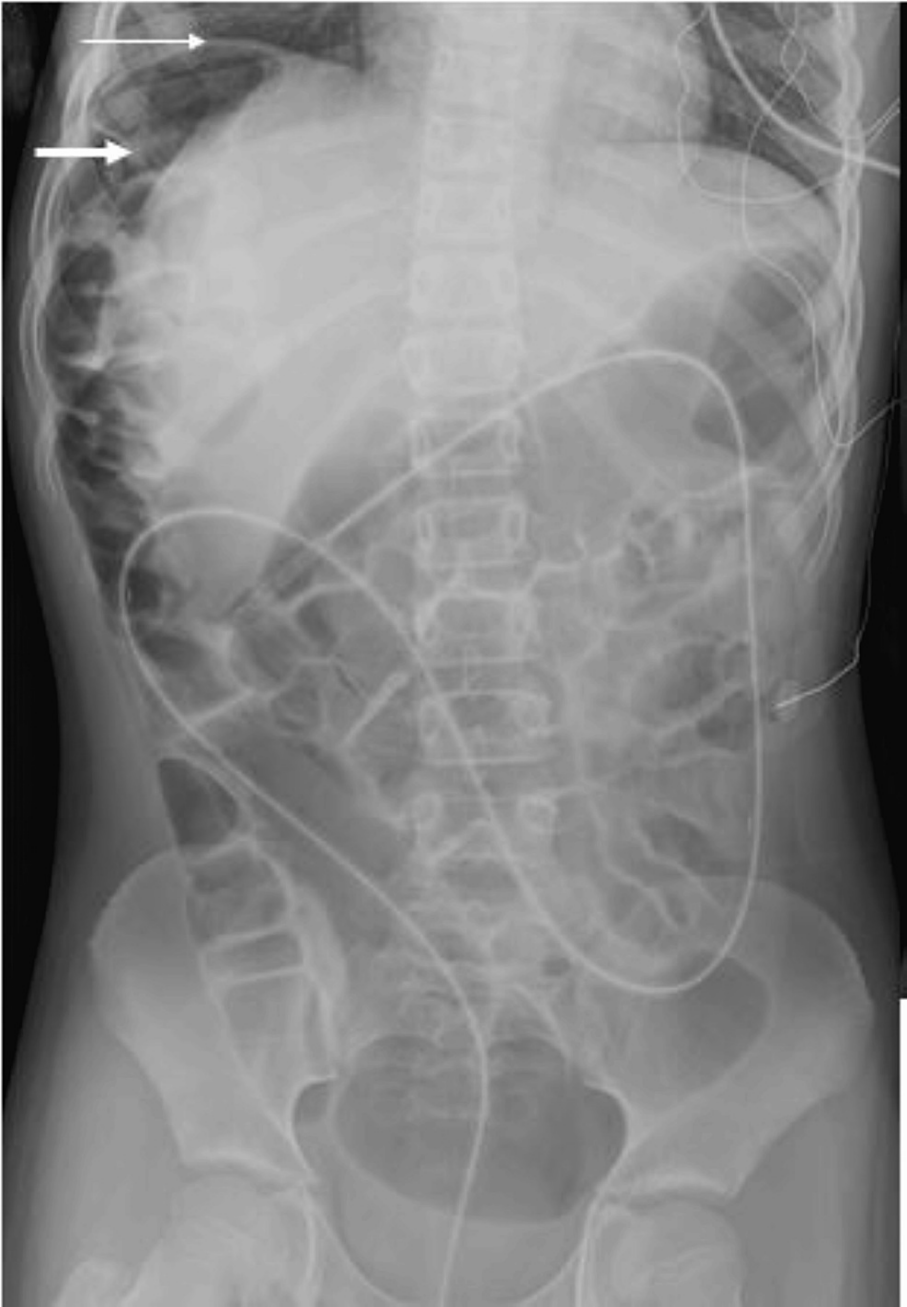
Supine abdominal radiograph The image is suggestive of the Chilaiditi sign including an elevated right hemidiaphragm (thin white arrow) with air distended bowel loop positioned between the liver and right hemidiaphragm. Note the prominent colonic haustral folds (thick white arrow) and intracolonic catheter

**Figure 2:**
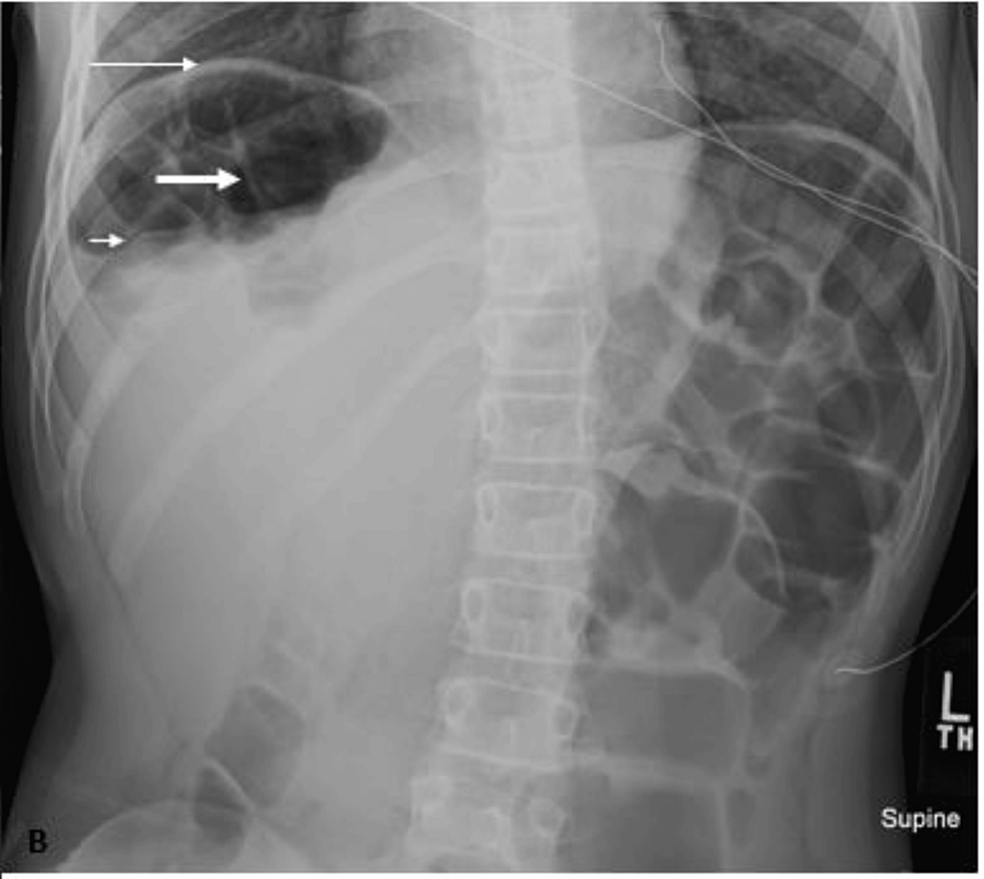
Supine abdominal radiograph 24 hours after Figure 1 The image shows multiple radiographic findings suggestive of the Chilaiditi sign including elevated right hemidiaphragm (thin white arrow) with air distended bowel loop positioned between the liver and right hemidiaphragm. Note the prominent colonic haustral folds (thick white arrow) and intracolonic catheter. The superior margin of the liver is depressed compared to the left hemidiaphragm (short white arrow)

**Figure 3:**
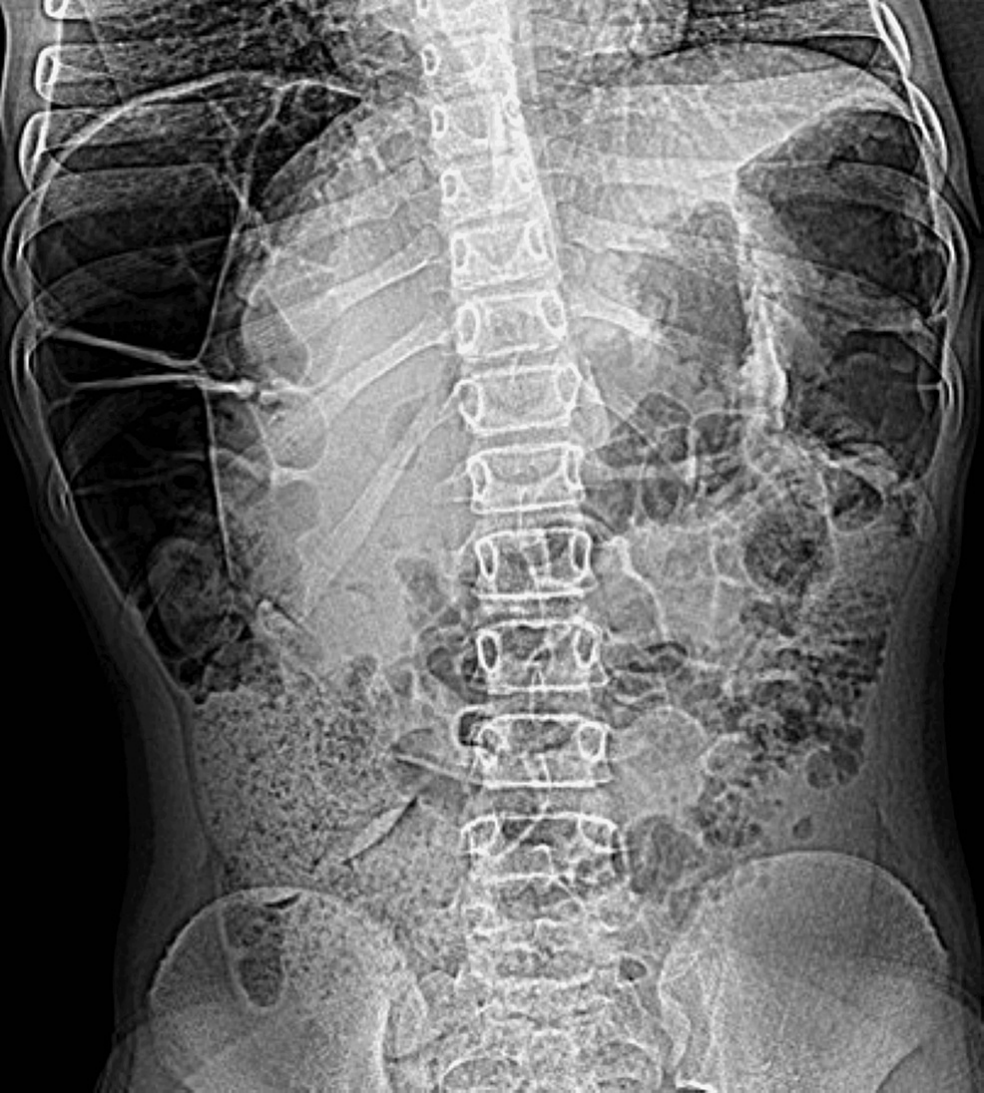
Supine abdominal radiograph from September 2021 The image showed radiographic findings favoring the Chilaiditi sign

**Figure 4:**
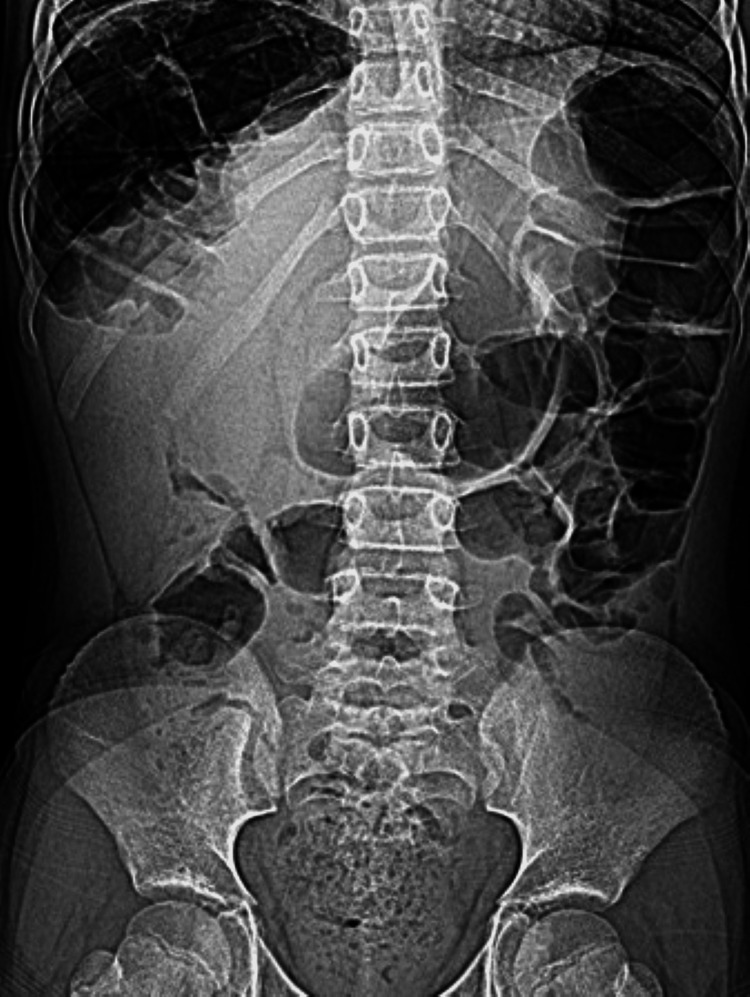
Supine abdominal radiograph from July 2021 The image showed radiographic findings favoring the Chilaiditi sign

**Figure 5:**
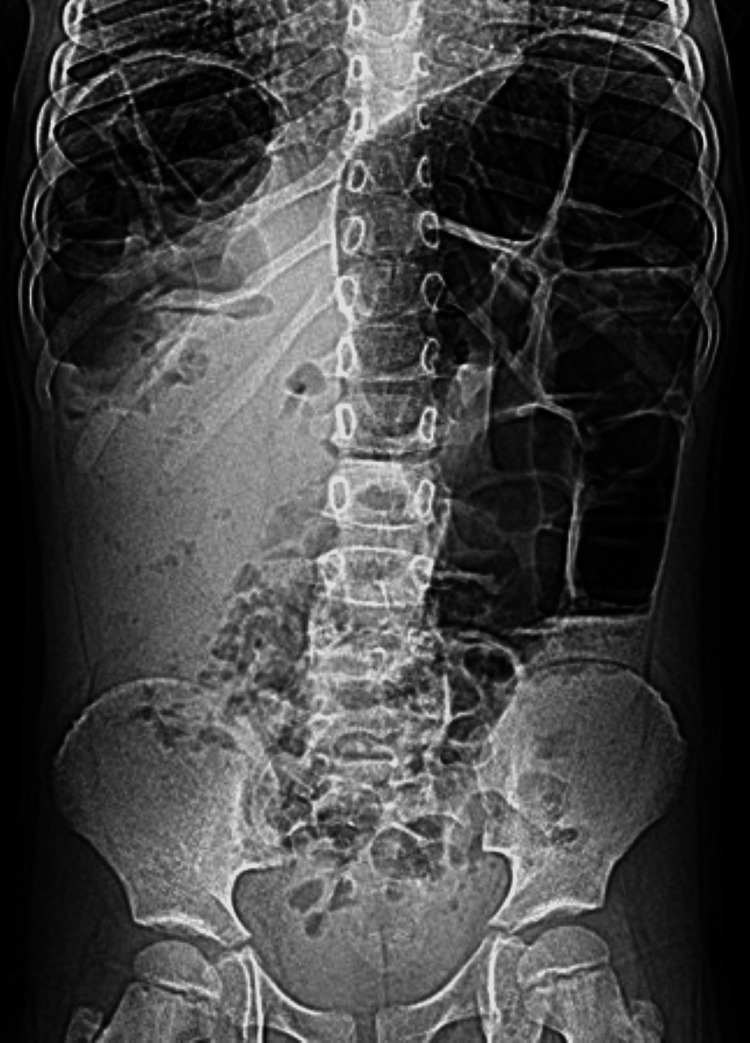
Standing abdominal radiograph from Dec 2017 The image showed radiographic findings favoring the Chilaiditi sign

A 10-year-old female with a history of autism, selective IgA deficiency, and constipation presented for colonoscopy, colonic and anorectal manometry, and anal onabotulinumtoxinA. She was found to have complaints of weight loss and constipation on presentation. An abdominal X-ray obtained showed bowel gas under the right hemidiaphragm along with a moderate amount of stool in the colon and colonic interposition between the diaphragm and the liver (Figures 1, 2), concerning for Chilaiditi sign. This abdominal X-ray was then compared to prior abdominal X-rays (Figures 3, 4, 5), which were also concerning for the Chilaiditi sign. She was managed with a bowel cleanout, with the completion of studies revealing colonic dysmotility, as well as compartmentalization of the sigmoid and rectum with the absence of coloanal reflex.

Case 2

**Figure 6:**
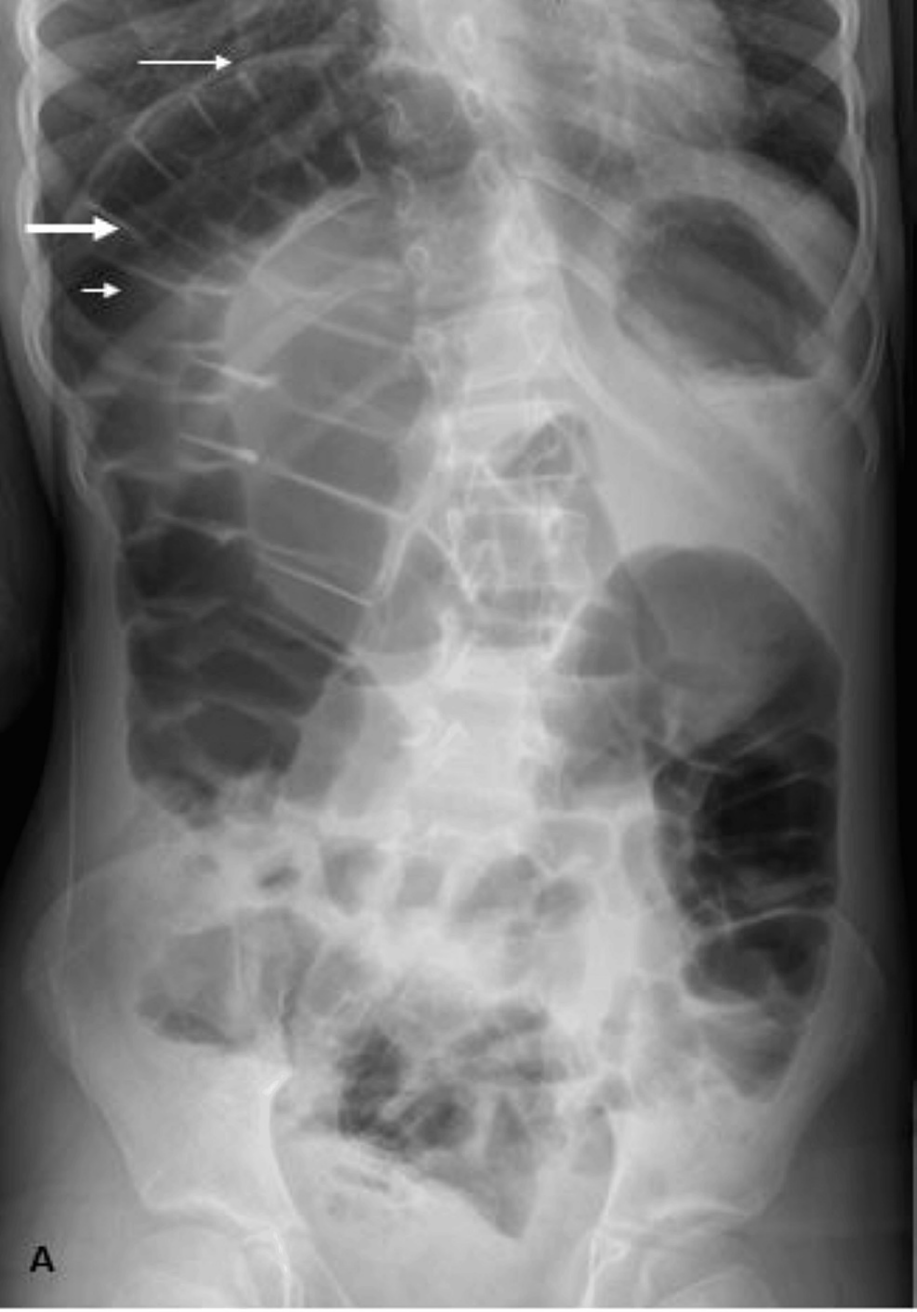
Standing abdominal radiograph The image showed significant elevation of the right hemidiaphragm (thin white arrow) with interposition of bowel loops between the liver and right hemidiaphragm. The bowel loops appear distended with air (pseudo-pneumoperitoneum sign). Note the presence of prominent incomplete haustral folds (thick white arrow) pointing to a large bowel loop. Remarkably, the superior margin of the liver appears lower compared to the level of the left hemidiaphragm (short white arrow). These radiographic findings are strongly suggestive of the Chilaiditi sign

**Figure 7:**
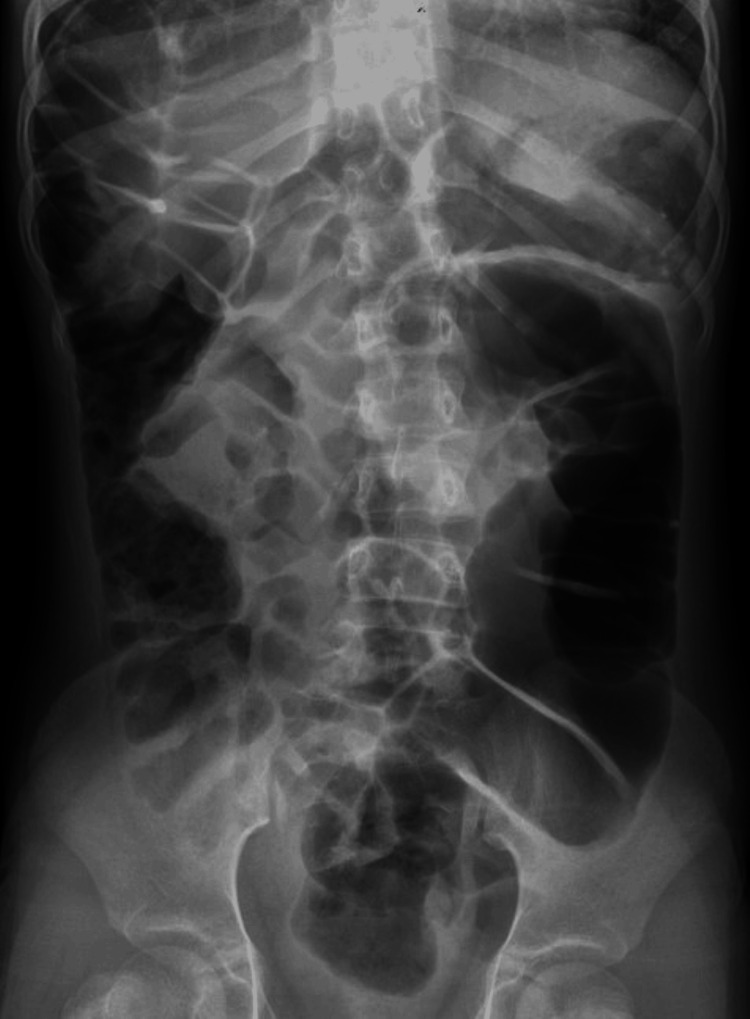
Supine abdominal radiograph acquired at the same time as Figure 6 The image demonstrated no significant change in the distribution of gas-filled bowel loops. This finding helps to distinguish the condition from true pneumoperitoneum where the air distribution is position-dependent

A nine-year-old female with a history of chronic constipation, developmental delay, dyskinesia, and hypotonia presented with abdominal pain, vomiting, constipation, and decreased appetite. Exam findings were notable for tachypnea, abdominal distension, and generalized abdominal tenderness. Abdominal X-ray displayed a dilated colon interposed between the liver and diaphragm and minimal formed stool in the colon (Figures 6, 7). Prior studies were reviewed and they showed significantly dilated and redundant sigmoid colon via barium enema and dyssynergia from hypotonia via anorectal manometry. The patient was managed with rectal irrigations and catheter decompression, which led to improvement of symptoms.

## Discussion

Chilaiditi sign is a radiological observation first identified by D. Chilaiditi in 1910. The sign is diagnosed when air is seen under the diaphragm and above the liver. This is typically the result of colonic interposition, commonly involving the right flexure of the colon. Chilaiditi syndrome refers to the condition when these findings are associated with symptoms, such as abdominal pain [5]. Chilaiditi syndrome is known as one of the many causes of pseudo-pneumoperitoneum. It is important to distinguish Chilaiditi syndrome from true pneumoperitoneum, as this diagnosis warrants immediate surgical intervention [10]. Raising awareness about this condition could aid in reducing the incidence of unnecessary exploratory surgeries due to misdiagnosed surgical emergencies [5].

Chilaiditi syndrome in pediatric patients is known to present with respiratory distress, vomiting, abdominal pain, abdominal distention, or constipation. The most common predisposing condition for Chilaiditi syndrome is aerophagia, often seen in children with developmental delay [3]. Chilaiditi sign and syndrome are seen in patients with disruption of normal diaphragmatic and abdominal anatomy, as well as in patients with laxity of suspensory ligaments, elevation of hemidiaphragm, reduced liver volume, obesity, and phrenic nerve palsy [2,6]. The etiology of Chilaiditi syndrome is not fully understood although many theories have been postulated; however, all theories related to its etiology describe decreased colonic motility as a fundamental cause [2,3,5]. Chilaiditi syndrome has been commonly seen in individuals with pancreatic cancer, Rett syndrome, lung cancer, systemic sclerosis, hemihypertrophy, and long-term use of antipsychotic medications [11].

Clinical observation is considered the treatment of choice in patients presenting with the Chilaiditi sign. Conservative treatment is regarded as the initial treatment of choice for patients with Chilaiditi syndrome. Conservative treatment includes bed rest, laxatives, decompression, and parenteral fluid therapy [7]. Surgical treatment is warranted in individuals with signs of bowel obstruction or ischemia, as well as in patients with recurrent Chilaiditi syndrome [2]. Surgical treatment typically consists of pinning the colon to the peritoneum, typically at the level of the umbilicus. Colonoscopy during initial examination or treatment is not recommended as there is a high risk of bowel perforation [7].

In our first case, the Chilaiditi sign was an incidental finding on the abdominal X-ray. The patient's presentation fit with the described associations in the literature: chronic constipation and developmental delay [2,3]. However, this patient did not have aerophagia or obesity, which are common findings in pediatric patients with Chilaiditi sign or syndrome [3]. This atypical finding on routine abdominal X-rays led to a follow-up X-ray. Since this patient was asymptomatic and there were no signs of bowel perforation or obstruction, the decision was made to continue with conservative management and clinical observation. It is important to note that this patient was admitted for a bowel cleanout and had been treated with different bowel regimens for her chronic constipation prior to admission. It is unknown if this patient had any symptoms that were not documented, warranting a diagnosis of Chilaiditi syndrome that had resolved by the time this patient was admitted.

In our second patient, Chilaiditi syndrome was a finding based on imaging, which had been prompted due to her symptoms of abdominal pain, emesis, and decreased appetite on presentation. This patient’s presentation was consistent with the described associations in the literature: hypotonia, chronic constipation, and developmental delay [2,3]. However, she did not have aerophagia and obesity, which, as already stated, are common findings in pediatric patients with Chilaiditi syndrome or sign [3]. Based on this patient’s symptomatic presentation, her imaging findings were identified as suggestive of Chilaiditi syndrome instead of Chilaiditi sign. The gaseous findings under the diaphragm were associated with Chilaiditi syndrome and a surgical emergency was ruled out. Hence, this patient did not receive any unnecessary surgical intervention. She was managed conservatively during both of her admissions and experienced symptom relief on discharge on both occasions. If the patient had any signs of bowel obstruction or perforation, management should have consisted of surgical intervention, instead of conservative management. Furthermore, if this patient continues to have recurring Chilaiditi syndrome, surgical intervention may be considered [2].

## Conclusions

Chilaiditi sign and Chilaiditi syndrome are relatively uncommon entities. Their prevalence among the general population is rare, and it is even rarer in the pediatric population. We believe this case series contributes to providing clinical awareness of these rare pediatric patient presentations, which would help avoid unnecessary invasive interventions. These reports also reassure clinicians in terms of their medical decisions to provide conservative management for these patients.
